# A Changing Perspective on the Role of Neuroinflammation in Alzheimer's Disease

**DOI:** 10.1155/2012/495243

**Published:** 2012-07-15

**Authors:** Donna M. Wilcock

**Affiliations:** Department of Physiology, Sanders-Brown Center on Aging, University of Kentucky, Lexington, KY 40536, USA

## Abstract

Alzheimer's disease (AD) is a complex, neurodegenerative disorder characterized by the presence of amyloid plaques and neurofibrillary tangles in the brain. Glial cells, particularly microglial cells, react to the presence of the amyloid plaques and neurofibrillary tangles producing an inflammatory response. While once considered immunologically privileged due to the blood-brain barrier, it is now understood that the glial cells of the brain are capable of complex inflammatory responses. This paper will discuss the published literature regarding the diverse roles of neuroinflammation in the modulation of AD pathologies. These data will then be related to the well-characterized macrophage phenotypes. The conclusion is that the glial cells of the brain are capable of a host of macrophage responses, termed M1, M2a, M2b, and M2c. The relationship between these states and AD pathologies remains relatively understudied, yet published data using various inflammatory stimuli provides some insight. It appears that an M1-type response lowers amyloid load but exacerbates neurofibrillary tangle pathology. In contrast, M2a is accompanied by elevated amyloid load and appears to ameliorate, somewhat, neurofibrillary pathology. Overall, it is clear that more focused, cause-effect studies need to be performed to better establish how each inflammatory state can modulate the pathologies of AD.

## 1. Introduction

 Alzheimer's disease (AD) is defined as the presence of amyloid plaques composed of amyloid-beta (A*β*) peptide aggregates and neurofibrillary tangles composed of hyperphosphorylated and aggregated tau protein. Neuroinflammation has been known to be present in AD since the original description of the histopathology of AD by Alois Alzheimer in 1907, who described “gliose,” inflammation of the glia. Since then, it has been shown in numerous studies, both mouse and human, that glial cells respond to the presence of AD pathological lesions (plaques and tangles) by morphologically changing their characteristics, expressing numerous cell surface receptors and surrounding the lesions [1, 2]. The prevailing view of neuroinflammation in AD for many years has been that it is an on-off phenomenon that contributes to the cytotoxicity of AD lesions and therefore contributes to the neurodegeneration in AD [3]. It is only within the recent decade that neuroinflammation has come to the forefront of AD research, not only with respect to its contribution to the neurodegenerative process, but also for its role in the clearance of AD lesions and beneficial contribution to AD progression. The dichotomy of the research findings on the role(s) of neuroinflammation in AD may be explained by the capacity of glial cells to generate multiple distinct phenotypes dependent upon the stimuli present.

 Glial cells describe the nonneuronal cells of the brain and include microglia, astrocytes, oligodendrocytes, and pericytes. These accessory cells are critical for the maintenance of an appropriate environment in which the neuronal cells can function optimally. This includes, but not limited to, ionic and osmotic homeostasis, myelination, debris removal, and neurotransmitter uptake and recycling. All glial cells are capable of achieving some degree of inflammatory response; however, the key cell type for the initiation, regulation, and resolution of the inflammatory response is considered to be the microglial cell. Derived from the macrophage cell lineage, microglia are specialized tissue macrophages in the brain and are capable of a broad range of inflammatory responses dependent upon the stimulus. This paper will summarize the published literature regarding different activation states of microglia and their subsequent impact on AD pathologies. This paper will then discuss how the field can use data from the peripheral macrophage body of literature to better characterize microglial activation states and begin to predict what impact each state will have on the progression of AD.

## 2. Microglia in AD

 For many years the body of literature regarding microglial cells and their role in AD focused on the negative influence inflammation would be thought to have on the progression of AD. This primarily focused on the concept of the autotoxic loop. Described in 1998 by E. G. McGeer and P. L. McGeer as a “vicious cycle,” the autotoxic loop is the description of the microglial activation in response to cellular debris in the AD brain, this microglial activation is then thought to result in the release of cytotoxic cytokines that then leads to a more rapid neuronal death, thus providing more cellular debris to further accelerate this process [3]. Evidence for this process stemmed from the finding that there are increased cytokine levels in the brains and CSF of AD patients. These cytokines, primarily IL-1*β* and TNF*α*, are known to be toxic to cells in culture and also toxic in the brain if injected into the brain parenchyma (reviewed in [4]). While there has been some evidence for the presence of an autotoxic loop, much of the body of literature suggests that the levels of cytokines are not great enough, or sustained enough in the AD brain to cause significant neuronal damage. Attempts to recreate the autotoxic loop led to some surprising findings, mostly that the initiation of an inflammatory response in the brain often leads to the clearance of amyloid plaques in transgenic mouse models.

Table 1 summarizes some of the studies that have stimulated inflammatory responses. It is initially apparent in Table 1 that most studies that have stimulated inflammation and activated microglia result in reduced amyloid load and do not present evidence of exacerbated neuronal degeneration. These data do not disprove the autotoxic loop, instead they suggest a much greater complexity to the inflammatory response of the brain than originally considered possible.

Lipopolysaccharide (LPS) is a gram-negative bacterial cell-surface proteoglycan that stimulates an innate immune response. Injection of LPS into the brain parenchyma of aged APP/PS1 mice originally aimed at stimulating the autotoxic loop resulted in microglial activation and the rapid reduction of amyloid deposits in the brain [5]. A later study identified the types of microglial activation occurring with LPS in wildtype mice using intraparenchymal LPS injections over a time course of 1, 6, and 24 hours as well as an extended time course of 3, 7, 14, and 28 days [6]. Over this time course, it was found that most gene expression changes in inflammatory markers peaked around the 3-day time point and slowly declined to normal levels by 14 days. The inflammatory markers examined included TNF*α* and IL1*β* as well as Fc*γ* receptors and scavenger receptors. Histologically, the same report found that microglial expression of cell-surface proteins including complement receptor 3 (also known as CD11b), CD45, scavenger receptor A, and Fc*γ* receptors II and III also peak around three days and then decline; however, some markers did not decline to control levels. Importantly, performance of a similar time course in APP/PS1 mice demonstrated that the majority of amyloid removal occurred between the time zero and three days, a small further decrease occurred at 7 and 14 days, while amyloid levels, surprisingly, rebounded to near time zero levels by 28 days [7].

In contrast to the amyloid data, LPS injection into tau transgenic mice showed opposite effects. Intraparenchymal injection of LPS into the rTg4510 tau transgenic mice resulted in exacerbation of tau pathology seven days after the injection [8]. This was determined by examining several phosphoepitopes of tau as well as Gallyas's silver staining-positive neurofibrillary tangles. In addition to the standard microglial cell surface markers including CD45, this study identified additional markers of microglial activation stimulated by LPS; these were arginase 1 and YM1. The importance of these markers will be discussed later in this paper. Additionally, LPS injection into the 3XTg mouse model of amyloid and tau pathology exacerbated the tau hyperphosphorylation [9]. These data suggest that tau and amyloid pathologies have opposite responses to the same inflammatory stimuli, in this case LPS. Whether this is the case for all inflammatory stimuli remains to be determined; however, these data should provide significant caution to the extrapolation of findings in amyloid depositing mice to the overall condition of AD.

Anti-A*β* immunotherapy is a potential therapeutic approach to the treatment of AD that uses either a vaccination approach [10] or passive immunotherapy approach [11] to increase levels of circulating anti-A*β* IgG molecules. There have now been numerous studies showing that this approach significantly lowers amyloid pathology and enhances behavioral performance in amyloid depositing transgenic mice (reviewed in [12]). Importantly, we previously performed a series of studies showing that anti-A*β* antibodies stimulate an inflammatory response in the brain. This occurs whether the anti-A*β* antibodies are directly injected into the brain parenchyma [13] or systemically administered in a passive immunization protocol [14]. We were also able to show that inhibition of this inflammatory response attenuates the amyloid reductions significantly [15, 16]. In contrast to the LPS studies, we were able to show in a different transgenic mouse model, the APPSw/NOS2^−/−^ mice that develop amyloid and tau pathologies, that anti-A*β* immunotherapy is able to lower both amyloid and tau pathologies while improving behavioral performance [17]. We examined the breadth of the inflammatory response in both passively immunized APP transgenic mice and actively vaccinated APPSw/NOS2^−/−^ transgenic mice, and we found that anti-A*β* immunotherapy stimulates the gene expression of IL-1*β*, TNF*α*, and IL-6 while concomitantly reducing the expression of inflammatory markers associated with wound repair; YM1 and arginase 1 [18]. These data contrast with those found with LPS injection, where IL-1*β*, TNF*α*, YM1, and arginase 1 were all significantly elevated.

Genetic overexpression of individual inflammatory cytokines has yielded data similar to those observed with LPS and anti-A*β* immunotherapy. Increased expression of TGF*β* by astrocytes results in reduced amyloid deposition and increased microglial activation in APP amyloid depositing transgenic mice [19]. In addition, an interesting finding in this study showed that while parenchymal amyloid deposition decreased, vascular amyloid deposition (cerebral amyloid angiopathy (CAA)) increased in a correlative manner. We observed a similar phenomenon with the anti-A*β* immunotherapy passive immunization studies, where we found increased CAA despite significantly decreased parenchymal amyloid deposition [20]. The data from Wyss-Coray et al. would suggest that inflammatory mechanisms may at least in part, be responsible for the shifted distribution of amyloid from the brain parenchyma to the cerebrovasculature.

TNF*α* and IL-1*β* are considered the major proinflammatory cytokines and are studied as classical markers of neuroinflammation. Individually, both have been implicated in an autotoxic loop as both are capable of inducing cell death in vitro and in vivo. Yet, when these pathways are targeted in amyloid depositing transgenic mice the data show that these cytokine pathways may have some beneficial action by ameliorating amyloid deposition. One study that genetically deleted TNF receptors I and II in the 3XTg mouse model of amyloid deposition and tau pathology showed that blocking TNF*α* signaling actually increases amyloid deposition and tau pathology [21]. Increased expression of IL-1*β* in the hippocampus of APP/PS1 amyloid depositing transgenic mice by genetic means resulted in reduced amyloid deposition and enhanced microglial activation [22]. The author suggest that IL-1*β*-mediated activation of microglia is the mechanism for the reductions in amyloid deposition. However, in contrast to these studies, other studies have shown a clear relationship between IL-1*β* and neurodegeneration. In a similar way to the LPS studies, IL-1*β* has been shown to be responsible for tau hyperphosphorylation in an in vitro coculture system of microglia and neurons [23]. Also, a positive correlation was observed when examining IL-1*β* levels compared to neurodegeneration in the APPV717F transgenic mice [24]. Therefore, while IL-1*β* may ameliorate amyloid pathology, it seems that the same pathways may also enhance tau pathology and neurodegeneration.

The contrasting data in different mouse models, cell culture models and stimulating agents clearly paints the picture of a complex process, one that cannot simply be defined as neuroinflammation.

## 3. Peripheral Macrophage Inflammatory States

 Macrophages are circulating immune effector cells that are prodigious phagocytes essential for the clearance of cellular debris and invading pathogens. The macrophages monitor the tissue environment and respond rapidly to any perturbations that may occur. Both macrophages and microglia originate from bone marrow hematopoietic stem cells that undergo differentiation into monocytes. These monocytes then undergo further differentiation upon reaching their target tissue to become macrophages, of which there are several types based on their tissue occupancy, or microglia if they enter the brain (reviewed in [25]). Macrophages are well understood to generate a variety of responses dependent upon the stimuli they are presented with. For instance, the presence of interferon-*γ* (IFN*γ*) or TNF*α* from T cells, antigen-presenting cells, or natural killer cells will stimulate the macrophage to express secrete proinflammatory cytokines and produce oxygen and nitrogen radicals. This state is termed classically activated or M1-activated macrophages. The M1 state has high microbicidal activity and is important as a defense mechanism, yet can also cause damage to the host if not tightly regulated [26]. Indeed, classically activated macrophages are implicated in the development of autoimmune pathologies [27].

**Figure 1:**
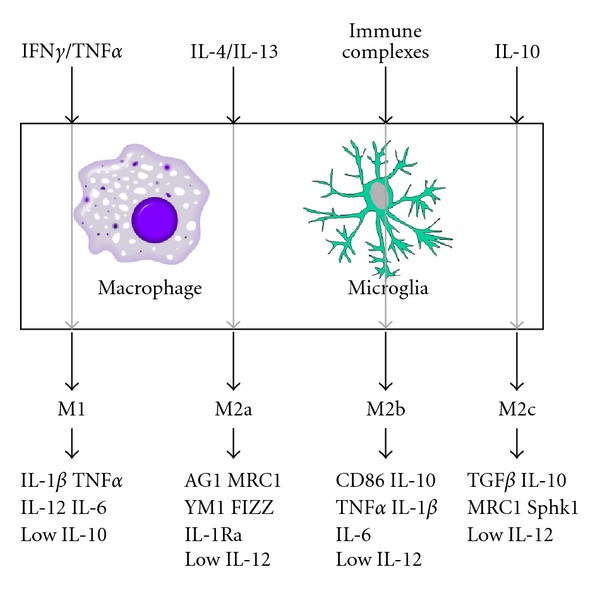
Schematic illustrating the M1, M2a, M2b, and M2c macrophage inflammatory states.

Stimulation of macrophages by IL-4 and/or IL-13 results in an M2a state, sometimes called a wound-healing macrophage [28]. The M2a macrophage state is characterized by high IL-1 receptor antagonist (IL-1Ra) and high arginase as well as expression of chitinases and other mediators that are known to contribute to the accumulation and reorganization of extracellular matrix [29]. The M2a responses are primarily observed in allergic responses, extracellular matrix deposition, and remodeling. The M2b macrophage state is stimulated by immune complexes (IgG antibody-antigen complexes), toll-like receptor activation, or IL-1 receptor ligands. This state is a combined M1 and M2a state, where arginase is high and IL-12 is low, but IL-1*β*, IL-6 and TNF*α* are also high. CD86 also appears to be a relatively specific marker for the M2b state [30]. Finally, the M2c macrophage state is stimulated by IL-10 and is sometimes referred to as a regulatory macrophage with anti-inflammatory activity [30, 31]. These cells express TGF*β* and high IL-10 as well as matrix proteins such as pentraxin and versican. The M2c state can also be generated through the hypothalamic-pituitary axis-derived glucocorticoids that inhibit the expression of pro-inflammatory cytokine genes and decrease the mRNA stability of these genes [31]. The M2c macrophages contribute to an environment that results in defective pathogen killing and enhanced survival of organisms. The macrophage states are summarized in Figure 1.

## 4. Applying the Macrophage Classification ****to Microglia and AD

**Figure 2:**
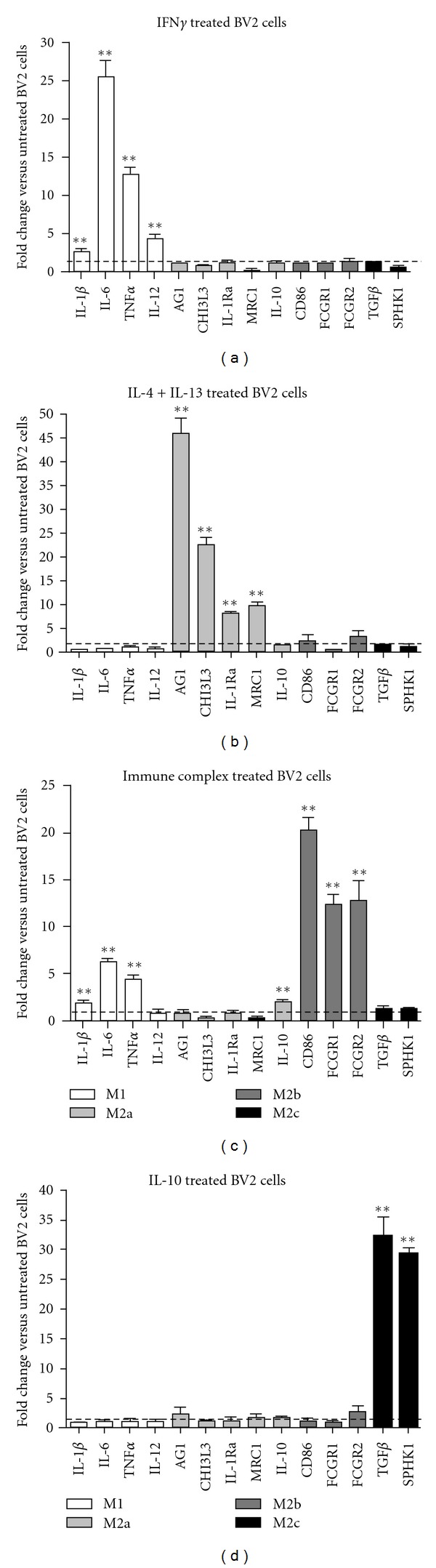
Stimulation of BV2 cultured microglial cells to polarize the response to M1, M2a, M2b, or M2c. BV2 microglial cells were cultured in normal DMEM media. When confluent, cell media was changed to serum-free DMEM media for 24 hours. Then media was changed to either DMEM plus IFN*γ* (2.5 ng/mL) to stimulate an M1 response (a), DMEM plus IL-4 (20 ng/mL) and IL-13 (20 ng/mL) to stimulate an M2a response (b), DMEM plus immune complexes prepared as described in [32] (5 *μ*g/mL A*β* coated with IgG) to stimulate an M2b response (c), DMEM plus IL-10 (10 ng/mL) to stimulate an M2c response (d), or DMEM alone to act as an untreated control. Cells were then harvested 12 hours after the start of treatment. We repeated the experiments 3 times, each on separate cultures of different passage numbers. Data are shown as fold change compared to untreated control BV2 cells. *P < 0.05  and **P < 0.01.

 Neuroinflammatory markers have been identified in the macrophage literature that can be applied to the study of microglia. Indeed, microglia are capable of expressing many of the macrophage markers identified in Figure 1. My laboratory, and others, has shown that the brains of amyloid-depositing mice, tau transgenic mice, and human AD expressing IL-1*β*, TNF*α*, IL-6, YM1, arginase 1, mannose receptor, TGF*β*, and IL-1Ra, among others. Most recently, my laboratory has performed in vitro studies on BV2 microglial cells to show that, given the correct stimulus, these microglial cells can generate very specific macrophage-like inflammatory responses using the M1, M2a, M2b, and M2c classifications.  Figure 2 shows the gene expression data obtained from BV2 microglial cells treated for 12 hours with IFN*γ* and TNF*α* to induce an M1 response, IL-4 and IL-13 to induce an M2a response, anti-A*β* IgG-A*β* immune complexes to induce and M2b response and, finally, IL-10 to induce an M2c response. As can be seen from the graphs in Figure 2, twelve hours of treatment of BV2 cells induced specific responses characterized by the expression of markers matching those described in the macrophage literature. These data suggest that microglia, given the correct stimuli, are capable of generating a range of responses similar to the macrophage. It will be important to follow up these studies in primary microglial cells to confirm that these findings are not unique to the immortalized BV2 microglial cell line.

My laboratory recently showed that passive immunization with anti-A*β* antibodies results in a shift in the inflammatory state of the brains of amyloid depositing transgenic mice. Tg2576 APPSw transgenic mice aged 18 months are normally biased to the M2a and M2c inflammatory states. Following only one month of weekly anti-A*β* antibody injections, the inflammatory state transitioned from M2a and M2c to M1; this was maintained following two and three months of administration [18]. Since the change occurred prior to significant reductions in amyloid deposition, it is likely that this inflammatory state transition is, at least partially, responsible for the reductions in amyloid due to the passive immunotherapy.

 The concept that M1 inflammatory state in the brain is associated with lower amyloid burden is supported by several studies that have examined components of this inflammatory state. For instance, IL-1*β* overexpression in the hippocampus of APP/PS1 transgenic mice results in decreased amyloid burden [22]. IL-1*β* is an M1 cytokine, so its overexpression may be reproducing the effect of an M1 neuroinflammatory state in the brain. Additionally, inhibition of TNF*α* signaling by the deletion of TNFRI and II in the 3XTg mice also showed reduced amyloid load [21]. Since TNF*α* is another M1 cytokine, the deletion of its signaling represents an artificial suppression of the M1 inflammatory state. Finally, LPS stimulates the secretion of IL-1*β*, TNF*α*, and IL-6; all M1 cytokines [6, 33]. LPS has been shown in several studies to significantly lower amyloid load in APP/PS1 transgenic mice.

The influence of other inflammatory states (M2) on amyloid load is less established. Few studies have directly targeted any of the M2 inflammatory pathways to establish cause-effect relationships between these states and amyloid deposition. The overexpression of TGF*β* may represent a bias toward the M2c inflammatory state however, in the absence of a characterization of the inflammatory milieu in these mice it is impossible to draw conclusions. It is apparent, however, from studies performed in aged transgenic mice that as amyloid accumulates in the brains of mice, the inflammatory state becomes increasingly polarized to the M2a inflammatory state. Also, my laboratory recently showed that lithium treatment enhances the M2a and M2c inflammatory phenotypes in APPSwDI/NOS2^−/−^ transgenic mice and increases amyloid deposition in the absence of changes in total A*β* measured biochemically. Until further studies are performed that directly enhance the M2a state using agents identified in the macrophage literature the exact relationship between the M2a state and amyloid deposition will remain unknown.

The relationship between neurofibrillary tangle pathology and the inflammatory state of the brain is relatively understudied in comparison to amyloid pathology. The few studies that have been done to establish relationships of hyperphosphorylated tau and neuroinflammation would suggest an opposite relationship to that of amyloid and neuroinflammation. Where M1 inflammatory phenotypes appear to ameliorate the amyloid pathology in numerous studies, induction of M1 phenotypes in tau transgenic mice or cell culture results in the exacerbation of tau pathology. LPS injection into the brains of rTg4510 tau transgenic mice has shown that the tau hyperphosphorylation and neurofibrillary tangle pathology are increased due to the LPS. Importantly, this study used an identical protocol to that used in APP/PS1 mice to show amyloid reductions due to LPS. Also, LPS injection in the 3XTg model of amyloid and tau pathology showed exacerbation of tau hyperphosphorylation after LPS injection [9]. Additionally, IL-1*β* treatment of microglia/neuron cocultures results in significant hyperphosphorylation of tau protein in the neuron [23]. Since IL-1*β* is an M1 cytokine, this again suggests that the M1 inflammatory state worsens the tau pathology associated with AD. Finally, my laboratory showed that biasing APPSwDI/NOS2^−/−^ mice to the M2a state ameliorates tau hyperphosphorylation normally present in these mice.

 Little has been studied in the human AD brain with respect to neuroinflammatory profiles, where a complete spectrum of M1 and M2 inflammatory markers has been examined. It has been shown that human AD brain is capable of expressing an array of inflammatory markers spanning the M1, M2a, M2b, and M2c inflammatory states [34]. Additionally, data from the ADAPT clinical trial that studied the preventative properties of nonsteroidal anti-inflammatory drugs (NSAIDs) in AD suggest that neuroinflammation may be complex and variable in the human population, since a subset of patients responded well to NSAIDs, while others declined more rapidly in response to the same NSAID [35]. Also, immunotherapy trials continue to show amyloid reductions in humans, while tau pathology remains relatively unchanged [36]. Future studies in humans should be focused on identifying the relationship between the pathologies of AD and the neuroinflammatory states.

## 5. Summary and Conclusions

 In summary, this paper has described that the macrophage inflammatory states of M1, M2a, M2b, and M2c, that are extremely well characterized in the immunology field, can be applied when examining the inflammatory state of the brain. By applying these states to the body of literature on the role of neuroinflammation in AD, the field can begin to establish cause-effect relationships for each neuroinflammatory state. It can be concluded that neuroinflammation is a complex, diverse process that can be characterized by examining a profile of markers associated with distinct inflammatory states. Finally, it is essential that more attention be focused on identifying relationships between each inflammatory state and each AD pathology so that the AD field can better target in a more directed, personalized manner to therapeutically treat AD.

## Figures and Tables

**Table 1:** Summary of some transgenic mouse studies that have modulated inflammation and the effects these modulations had on the pathology.

Mode of inflammatory modulation	Genetic model of AD	Pathological changes observed				References
		Amyloid load	Tau pathology	Neuronal degeneration	Microglial “activation”
LPS intracranial	APP/PS1 amyloid	↓			↑	[5, 7]
LPS intracranial	rTg4510 tau		↑		↑	[8]
Anti-A*β* immunotherapy	APP amyloid	↓			↑	[13, 14]
Anti-A*β* immunotherapy	APP/NOS^−/−^ amyloid, tau, neuron loss	↓	↓	↓	↑	[17]
IL-1*β* overexpression in brain	APP/PS1 amyloid	↓			↑	[22]
TGF*β* overexpression in brain	APP amyloid	↓			↑	[19]
TNFR1 and R2 deletion	3Xtg amyloid and tau	↑			↓	[21]
